# How Much Overtesting Is Needed to Safely Exclude a Diagnosis? A Different Perspective on Triage Testing Using Bayes' Theorem

**DOI:** 10.1371/journal.pone.0150891

**Published:** 2016-03-03

**Authors:** Jonne J. Sikkens, Djoeke G. Beekman, Abel Thijs, Patrick M. Bossuyt, Yvo M. Smulders

**Affiliations:** 1 Department of Internal Medicine, VU university medical centre, 1081HV Amsterdam, the Netherlands; 2 Department of Clinical Epidemiology, Biostatistics and Bioinformatics, Academic Medical Centre Amsterdam, 1105 AZ Amsterdam, the Netherlands; Instituto de Higiene e Medicina Tropical, PORTUGAL

## Abstract

Ruling out disease often requires expensive or potentially harmful confirmation testing. For such testing, a less invasive triage test is often used. Intuitively, few negative confirmatory tests suggest success of this approach. However, if negative confirmation tests become too rare, too many disease cases could have been missed. It is therefore important to know how many negative tests are needed to safely exclude a diagnosis. We quantified this relationship using Bayes’ theorem, and applied this to the example of pulmonary embolism (PE), for which triage is done with a Clinical Decision Rule (CDR) and D-dimer testing, and CT-angiography (CTA) is the confirmation test. For a maximum proportion of missed PEs of 1% in triage-negative patients, we calculate a 67% 'mandatory minimum' proportion of negative CTA scans. To achieve this, the proportion of patients with PE undergoing triage testing should be appropriately low, in this case no higher than 24%. Pre-test probability, triage test characteristics, the proportion of negative confirmation tests, and the number of missed diagnoses are mathematically entangled. The proportion of negative confirmation tests—not too high, but definitely not too low either—could be a quality benchmark for diagnostic processes.

## Introduction: A Clinical Vignette

Imagine overhearing the following discussion: Two physicians discuss their experience with CT-angiography (CTA) for diagnosing pulmonary embolism (PE). Dr. A says: “In our hospital we were interested in the quality of our diagnostic work-up for PE. We found that 80% of CTA´s performed for this indication were normal”. “Really?” replies dr. B. “In our hospital we have a negative CTA proportion of only 30%. It appears to me you are ordering way too many CTAs”. “Hmm, I don't think it is a question of too many scans ordered”, replies dr. A. “In fact, I think your low proportion of negative scans mathematically translates into a higher risk of missing PE. In contrast, my proportion of negative scans might well be in perfect concordance with an appropriately lower proportion of missed PEs.”

## The Problem

The imaginary discussion addresses a common clinical situation, in which severe disease needs to be ruled out, but the confirmation test is expensive, troublesome, or poses risks: the possible consequences of radiation exposure in our example. In such cases, it is common to pre-select patients for the confirmation test on the basis of a prior test: mammography before biopsy, abdominal imaging before exploratory laparotomy or, as in this example, a clinical decision rule (CDR) combined with D-dimer testing prior to a CTA for PE.

The issue between the discussants in the vignette centers around the question whether a given proportion of negative CTAs can be linked to a specific PE proportion in triage-negative patients—false negatives—when triaging is based on both the CDR and D-dimer. A similar example, however, might just as well have applied to a surgeon who claims to 'rarely have a negative laparotomy', but also to 'never miss acute appendicitis'. Similar to dr. B in the vignette, many professionals, but also insurance companies negotiating over reimbursement, may associate a high number of negative CTAs with wasteful and potentially harmful overuse of resources. However, perhaps these negative CTAs are necessary to maintain a low proportion of missed PEs?

It helps to phrase the question in more general terms: Is there a mathematical relationship between the proportion of negative confirmation tests and the number of missed diagnoses? We propose there is, and that this can be calculated using the well-known principles of Bayesian reasoning.[1,2] In other words, we take a new perspective on an old method. We will explain that the proportion of negative confirmation tests can serve as a diagnostic quality benchmark, and that a low proportion of negative confirmation test results may quite precisely reflect an unacceptably high risk of missing diagnoses. We stick with the example of PE, but the general principles and mathematics apply to any disease and diagnostic strategy. In the online supplement, we provide software tools to perform the calculations for any clinical situation.

## The Example: Pulmonary Embolism (PE)

**Fig 1:**
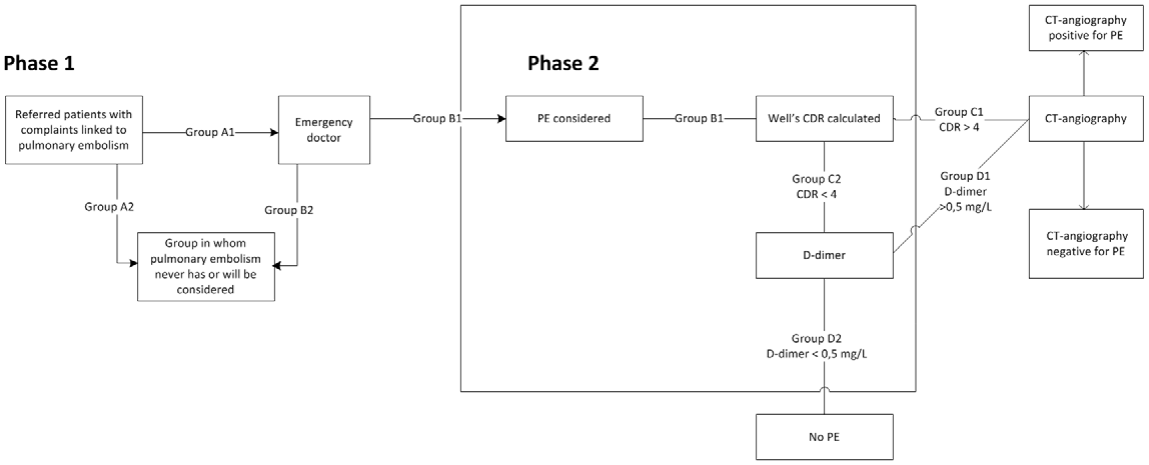
Process of pulmonary embolism diagnosis. Phase 1. The outpatient must be selected to be send to the ER. Phase 2. The ER doctor must think of PE before starting diagnostic work up. This highly depends on his/her clinical intuition. This is referred to as “the clinical suspicion”, which is the average of all ER doctor’s combined. Phase 3. Diagnostic work-up depends on the characteristics of the tests. CDR: clinical decision rule.

A missed diagnosis of PE can lead to severe morbidity or even death. Any diagnostic strategy therefore aims to safely exclude the disease.[3,4] A negative CTA does exactly that, but is too cumbersome, expensive, and harmful in terms of radiation exposure to perform in anyone with clinical suspicion. Therefore, many hospitals use a diagnostic procedure that includes the use of a CDR and D-dimer testing as a first procedure to select patients for CTA, which nowadays is considered the reference test for ruling out PE. We will now describe the procedure step-by-step (Fig 1).

Phase 1: Selection of patients (A1) preselected by a general physician and/or an emergency room (ER) nurse who will be presented to the ER physician with any combination of complaints possibly pointing to PE. This group is examined by the ER physician who immediately rejects (or fails to consider) the possibility of PE in some patients (B2) and considers the diagnosis in others (B1). Note that this is a very implicit, and partly subconscious, diagnostic process: no explicit guideline or algorithm helps a clinician to decide whether the diagnosis of PE should be considered in the first place. In other patients (A2) PE will never be considered; in this last group patients with PE remain undiagnosed and will thus be missed.

Phase 2: The group in whom PE is formally considered ('PE considered'; group B1) will proceed to undergo the diagnostic procedure of CDR and D-dimer testing. The actual proportion of patients with PE in this group depends on the population prevalence modified by how well patients are pre-selected for this group. We call this proportion the pre-test PE probability: the proportion of patients with PE in those undergoing triage testing.

After performing the CDR in this group, all patients scoring above the CDR’s cut-off point will proceed to undergo CTA (C1). In all other patients (C2), a D-dimer test is performed. Patients with elevated (>0.5 mg/L) D-dimer test results (D1) also undergo CTA, while in the remaining patients (D2) the diagnosis of PE is ruled out. For the Wells CDR, a cut-off score of 4 or less with a normal D-dimer test (<0.5 mg/L) has shown an acceptable 3-month proportion of missed PEs of 0% to 0.9%, with a 95% confidence interval (CI) upper limit of 2.7%,[3–9] For this example, we used the test characteristics of this diagnostic algorithm as estimated in a large prospective cohort study, which reported a sensitivity and specificity of 98.8% (95% (CI): 96.9–99.7%)and 37.5% (95%CI: 34.7–40.4%), respectively.[9] This high sensitivity is appropriate in view of the primary concern of the physician, which is not to miss cases of PE. Please note, throughout the article we used the test characteristics of the combination of tests described above and in Fig 1, rather than the individual characteristics.

If we assume the maximum percentage of missed PEs within phase 2 after completion of the diagnostic evaluation (false negatives) to be no higher than 1%, what is the corresponding number of negative CTAs? And what is the corresponding pre-test probability?

## The Solution: Taking Bayes’ Theorem Back and Forth

According to Bayes' theorem, multiplying the pre-test odds by the likelihood ratio of a diagnostic test results in the post-test odds (see Box 1).[1] The likelihood ratio can be estimated from the test sensitivity and specificity. The pre-selection process as described in Fig 1 (phase 1) determines the value of the pre-test PE probability, resulting in a group of patients in which the diagnosis of PE is considered (B1).

**Box 1. Calculation of Bayesian parameters.**

**Pre-test probability:** the proportion of patients undergoing triage testing who have the target disease (also referred to as the disease prevalence in those being tested)

**Positive likelihood ratio (LR+) calculation**: sensitivity/(1-specificity)

**Negative likelihood ratio (LR-) calculation:** (1-sensitivity)/specificity

**Positive predictive value (PPV):** proportion of patients with the target disease in those testing positive

**Proportion of false negatives:** proportion of patients with the target disease in those testing negative (one minus negative predictive value).

**Negative predictive odds calculation:** pre-test odds x negative likelihood ratio.

**Conversion of odds to probability:** odds/(odds+1)

**Conversion of probability to odds:** probability /(1- probability)

Assuming a maximum allowed proportion of missed PEs of 1% within phase 2, we now use Bayes' theorem to calculate the corresponding pre-test PE probability (Table 1A). This 1% proportion of missed PEs equals posterior-test odds of 0.01, using the probability-to-odds conversion method (see Box 1 for all formulas). These odds can in turn be divided by the negative likelihood ratio, which results in the corresponding prior odds (0.32), which can then be converted to the prior PE risk of 24%. This implies that if 24% of inpatients entering the CDR/D-dimer triage strategy have PE, the diagnosis can be excluded with acceptable certainty. If fewer patients being tested actually have PE, the negative predictive value will be higher, and even less than 1% of those testing negative will have PE. If more patients being tested actually have PE, more than 1% of those testing negative will have PE.

**Table 1:** Relation between prior disease probability, proportion of negative confirmation tests, and proportion of missed diagnoses.

Fixed parameter	Prior probability ↔ prior odds x likelihood ratio = posterior odds ↔ posterior probability	Proportion of negative confirmation tests
**A 1% negative predictive value**	24% ← 0.32 = 0.01 / 0.032 (LR-) ← 0.01 ← **1%**	
	24% → 0.32 x 1.58 (LR+) = 0.50 → 33%	67%
**B 40% prevalence in those tested**	**40%** → 0.67 x 0.032 (LR-) = 0.021 → 2.09%	
	**40%** → 0.67 x 1.58 (LR+) = 1.05 → 51%	49%
**C 80% negative confirmation tests**	13.7% → 0.16 x 0.032 (LR-) = 0.005 → 0.50%	
	13.7% ← 0.16 = 0.25 / 1.60 (LR+) = 0.25 ← **20%**	80%

We assumed triage test sensitivity and specificity of 98.9% and 37.5%, respectively. Fixed parameters serve as starting points for the calculations and are indicated in bold numbers. Arrows indicate direction of calculation.

We can subsequently calculate the proportion of positive CTAs corresponding to this 24% pre-test probability, by multiplying it with the positive likelihood ratio of the triage strategy. This yields post-test odds of 0.50, leading to 33% positive predictive value. Hence, to rule out PE with a proportion of false negatives of 1%, the proportion of negative confirmation tests should not be lower than 67%. In several studies, the 3 month proportion of false negatives is reported around 0.5%, [3–8] which corresponds to a PE proportion of 14% in those tested, with an associated proportion of negative CTAs of at least 80%, roughly the same proportion as Dr. A reported in the clinical vignette.

## Effects of Changing the Parameters

What will happen if clinicians are more reluctant to enter patients into the diagnostic strategy, and the pre-test probability is higher than 24%? First, we can logically assume that the proportion of missed PEs in groups A2 and B2 will be higher with a higher proportion of clinical significant PE. Second, assuming identical CDR + D-dimer test characteristics, a higher pre-test probability will lead to fewer negative CTAs, but the risk of having PE after a low Wells CDR score and a negative D-dimer result will be higher than the acceptable minimum of 1% (Table 1B). This means the overall proportion of missed PEs will increase.

In all cases, when to consider PE is largely a subjective judgment. The actual proportion of negative CTAs, however, can easily be drawn from the hospital's administrative records. As just illustrated, Bayes' theorem can be used to derive, from the proportion of negative scans, the hospital’s pre-test probability (assuming the test characteristics apply in this hospital) and to a post-test probability and the proportion of false negatives (Table 1C). The pre-test probability reflects the clinical threshold clinicians apparently have when they decide to start the PE triage strategy. Higher pre-test probabilities correspond to a higher threshold for starting testing in patients with suspected PE.

**Fig 2:**
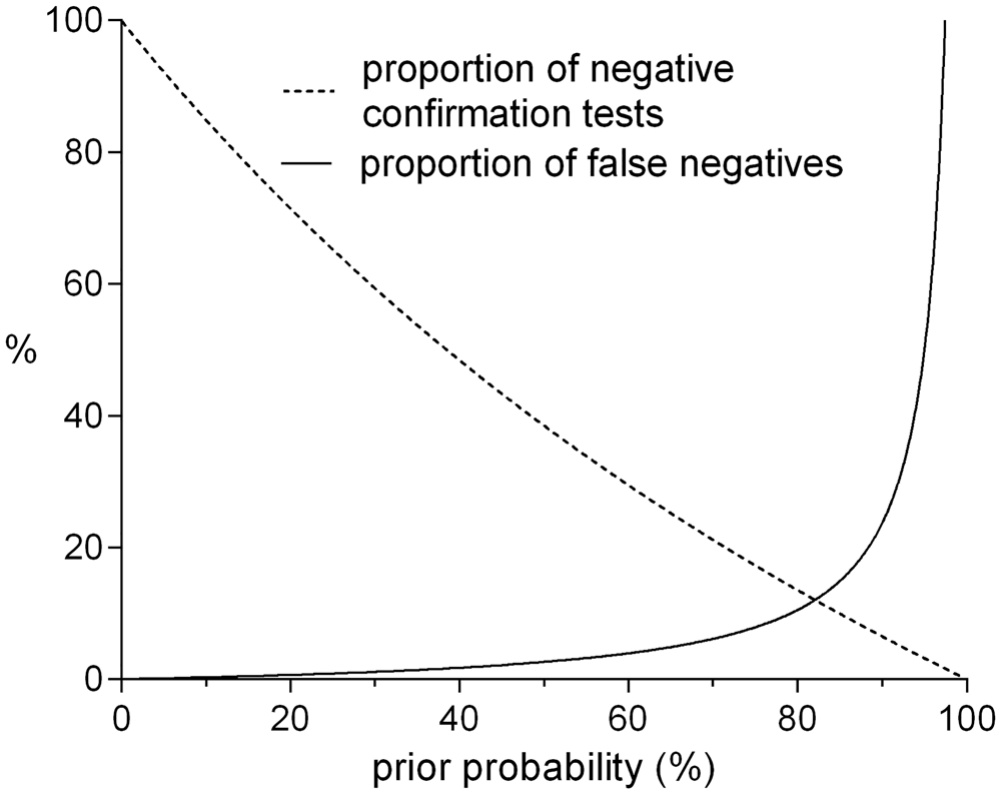
The relationship between the pre-test probability, the proportion of negative confirmation tests and the risk of missing a diagnosis in those testing negative. In the online version, sensitivity and specificity of the triage strategy (98.9% and 37.5%, respectively, in this example) can be varied, illustrating the profound impact this has on the other parameters. We here assume that sensitivity and specificity are constant across all possible pre-test probabilities.

The mechanism described above is illustrated in Table 1 for a triage strategy with a sensitivity of 98.9% and a specificity of 37.5%, as in the PE example. The online supplement provides tables in which each of the parameters can be varied, and the corresponding variables are calculated automatically. Fig 2 illustrates the mechanism graphically, and is also supplied online as a program that illustrates the influence of changes in sensitivity and specificity of the triage procedure on the associations between pre-test probability, proportion of negative confirmation tests, and the proportion of false negatives (missed diagnoses).

Going back to the clinical vignette, and entering the data presented into the calculation models, we obtain the following results: the triage strategy in the hospital with 80% negative CTAs leads to a maximum of 0.50% false negatives (Table 1C), while for the strategy in the hospital with 30% negative CTAs this is 4.51%; more than triple the risk which is generally considered acceptable. Dr. A. was clearly right!

It is not uncommon for diagnostic tests or algorithms to perform slightly worse in clinical practice than in clinical studies. What happens, for example, if sensitivity of the CDR+D-dimer triage strategy for PE would be 98% in clinical practice, rather than the 98.9% we used thus far? Maintaining a maximum allowed proportion of false negatives of 1%, the ‘mandatory minimum’ proportion of negative CTAs increases substantially from 67% to 87%. Combining the data from the clinical vignette with this slightly lower sensitivity, the triage strategy in the hospital with 30% negative CTAs leads to 7.35% false negatives, while for the hospital with 80% negative CTAs, this is 0.84%; still below the generally accepted maximum proportion of 1%.

As mentioned, we have thus far used test characteristics from a single large cohort study. To show how the principle described in this study translates to other situations we calculated the outcomes when applied to the test characteristics reported in a meta-analysis of a CDR and VIDAS-d-dimer-based algorithm.[10] This analysis reported a very high sensitivity of 99.7%, a specificity of 47.4% and a negative predictive value 99.9% (implying a proportion of missed PEs of 0.1%). We can then calculate that 77% of their CTAs were negative and that in the tested population, PE was present in only 14% of cases. Under these conditions, the percentage of negative CTAs could be decreased to 25% while still missing no more than 1% of PEs in those tested. To achieve this, the threshold for performing the diagnostic algorithm would have to increase to a pre-test PE probability of 61.5%.

It is often assumed that sensitivity and specificity are not influenced by the prevalence of disease in a specific population. However, these characteristics do in fact depend on context-specifics such as average disease severity, symptom reporting, and prevalence of other diseases with similar symptoms. To estimate the uncertainty associated with our outcomes, we performed a Monte Carlo simulation study by replicating the diagnostic study from which we used the test characteristics.[11] Based on the originally reported sensitivity, specificity and PE prevalence, we recalculated these estimates by drawing from binomial distributions in a population size equal to this source study, repeated 10.000 times.[9] The simulation was performed in STATA (version 12, 2013; College Station, TX), do-file available on request (j.sikkens@vumc.nl). This procedure revealed that the aforementioned ‘mandatory minimum’ percentage of 67% negative CTAs is tied to a 95% CI of 32% to 81%. Conversely, this percentage of 67% negative CTAs is tied to a proportion of 1% false negatives, with a 95% CI of 0.2% to 2.1%. For physician B from the vignette, the 30% CTAs lead to a proportion of 4.5% false negatives with a 95% CI of 1.0% to 9.2%.

## Discussion

To resolve the issue presented in the clinical vignette, we have used Bayes theorem. We demonstrate that, when using the common triage diagnostic algorithm for PE (CDR + D-Dimer), at least 67% of CTAs should be negative to ensure that no more than 1% of patients with a negative triage result have PE within phase 2 of the triage strategy. In more general terms, we have explained how the pre-test disease probability, the sensitivity and specificity of a triage strategy, the proportion of negative confirmation tests, and the risk of missing diagnoses are interrelated. This paper shows how proportions of negative confirmation tests can be used as an indication of the proportion of missed diagnoses, and how a ‘mandatory minimum’ of negative confirmation tests could be used as a benchmark for the quality of any diagnostic process. Importantly, our method can be utilized by anyone because it is based on the well-known Bayes theorem comprising mathematically simple calculations that can even be performed using a hand calculator.

We believe our findings are relevant to a wide variety of clinicians. They illustrate that an excessive urge to reduce the rate of negative confirmation tests, be it from a financial or from any other perspective, has a clear downside in terms of missed diagnoses. The decisive parameter is the clinical testing threshold in PE suspects (to enter phase 2, Fig 1): the strength of suspicion required before physicians decide to enter the patient in the diagnostic algorithm. In our example, this should correspond to a group level probability not exceeding 24%. Let’s assume that both physicians A and B were correctly using the same validated triage strategy (CDR + D-dimer). The different rates of negative CTAs then most likely imply that the threshold for considering PE and starting further evaluation (Fig 1, phase 1 & 2), differed between physicians A & B.

The level of the clinical threshold can differ due to differences in the presenting case mix, but is ultimately generated in the mind of the physicians (phase 1). It may also be related to differences in training, experience, personality ('defensive' physicians may consider pulmonary embolism more often in atypical patients), or even a subconscious reluctance to be confronted with the burden or costs of 'excessive rates of negative CTAs. Assuming the test characteristics of the triage strategy were identical to those reported in the literature, the pre-test PE probability in their patients having PE can be calculated, and would be 13.7% (Table 1C) and 59.6% for physicians A and B, respectively.

A recent meta-analysis found that, in daily practice, the clinical threshold physicians apparently have before they order a formal evaluation of PE corresponds to a pre-test (group level) probability ranging from 10% to 39%.[12] Based on our calculations, these probabilities translate into a proportion of negative CTAs between 85% and 50%, and a proportion of false negatives ranging between 0.35% and 2.00%, respectively. This variability underlines that the diagnostic process is highly inter-physician-dependent.

The process of initial selection for referral to the emergency room physician and his/hers subsequent selection for further testing is decisive for the total number of PE diagnoses that is missed (false negatives and missed diagnoses in patients not tested). Our calculations are based on a minimum acceptable risk of having PE after the formal triage algorithm results in a decision to discharge without CTA (false negatives). They do not apply, however, to the total number of missed PE diagnoses, which remains critically dependent on (1) the initial decision of who to refer to the hospital, and (2) who is subsequently selected by the emergency room physician for formal PE testing by CDR, D-dimer, etc. (Fig 1, group A2 & B2). The quality of this process is likely to be different between referring and emergency room physicians, but is almost impossible to grasp in numeric terms.

In this study, we considered a 1% proportion of missed PEs as an acceptable risk. This obviously is an arbitrary level, which should be weighed against the risks and costs of increased diagnostic testing. Advanced medical decision-making methods to deal with the relative costs of such competing risks are available in literature but these fall outside the scope of this article.[9]

In view of these considerations the proportion of negative confirmation tests (i.e. not too high, but certainly not too low) could be used as a quality benchmark for any diagnostic process. As the results of our simulation study show, the range of negative test proportions can be considerable, and even very high proportions cannot be directly interpreted as a sign of overtesting. Any inclination to change protocols or procedures in order to reduce negative confirmation test should be preceded by a decision on the maximum acceptable proportion of missed diagnoses, and calculation of the mandatory minimum proportion of negative confirmation tests needed not to exceed this proportion of missed diagnoses.

## Supporting Information

S1 Fig The relationship between the percentage of pre-test probability, the proportion of negative confirmation tests and the proportion of missed pulmonary embolisms. Sensitivity and specificity of the screening strategy (99% and 38%, respectively, in this example) can be varied using the slides or by changing the numbers in the yellow area. This illustrates the profound impact this has on the other parameters.
(XLS)
